# SCD1 is required for EGFR-targeting cancer therapy of lung cancer via re-activation of EGFR/PI3K/AKT signals

**DOI:** 10.1186/s12935-019-0809-y

**Published:** 2019-04-18

**Authors:** Kelin She, Shenghua Fang, Wei Du, Xingxing Fan, Jiaxi He, Hui Pan, Liyan Huang, Ping He, Jun Huang

**Affiliations:** 1 Department of Thoracic Surgery, The First Affiliated Hospital of Guangzhou Medical University, Guangzhou Research Institute of Respiratory Disease, China Key Laboratory of Respiratory Disease, National Center for Clinical Trials on Respiratory Diseases, No. 151 Yanjiangxi Road, Guangzhou, Guangdong 510120 China; 2 The Central Hospital of Shaoyang City, Shaoyang, Hu’nan China; 3 Affiliated Cancer Hospital & Institute of Guangzhou Medical University, Guangzhou, China; 4 Department of Thoracic Surgery, Dongguan People’s Hospital, Dongguan, Guangdong China; 5 State Key Laboratory of Quality Research in Chinese Medicine, Macau Institute for Applied Research in Medicine and Health, Macau University of Science and Technology, Macau, SAR China; 6 Department of Pathology, The First Affiliated Hospital of Guangzhou Medical University, Guangzhou, China

**Keywords:** SCD1, EGFR, AKT, EMT, Lung cancer

## Abstract

**Background:**

Cancer cells are characterized by aberrant activation of lipid biosynthesis, producing saturated fatty acids and monounsaturated fatty acids via stearoyl-CoA desaturases (SCD) for regulating metabolic and signaling platforms. SCD1 overexpression functions as an oncogene in lung cancer and predicts a poor clinical outcome. This study aimed to investigate the role of SCD1 inhibition by EGFR inhibitor (Gefitinib)-based anti-tumor therapy of lung cancer both in vitro and in vivo.

**Methods:**

CCK-8 assay was performed to determine cell viability. The SCD1 mRNA level was detected by qPCR. The protein levels were assessed by Western blotting. E-cadherin and N-cadherin levels were determined by immunofluorescence. Apoptosis detection was conducted by flow cytometry. Cell migration or invasion was evaluated by transwell assay. The tumor sizes and tumor volumes were calculated in nude mice by subcutaneous injection of A549 cells transfected with vector of pcDNA3.1-SCD1 or negative control. Expression of Ki-67 was detected by immunohistochemistry.

**Result:**

SCD1 up-regulated expression was observed in lung cancer cell lines. Cells with overexpressed SCD1 had high IC50 values for Gefitinib in A549 and H1573 cell lines. Overexpression of SCD1 inhibited Gefitinib-induced apoptosis, decreased cell vitality and impaired ability of migration and invasion, while these effects were counteracted by A939572. Mechanistically, SCD1 promoted the activation of proliferation and metastasis-related EGFR/PI3K/AKT signaling, and up-regulated epithelial to mesenchymal transition (EMT) phenotype in the two cell lines, which was restored by SCD1 inhibition. Furthermore, in spite of EGFR inhibition, overexpression of SCD1 in vivo significantly promoted tumor growth by activating EGFR/PI3K/AKT signaling in tumor tissues, but A939572 treatment restricted SCD1-induced tumor progression and inhibited EMT phenotype of cancer cells in vivo.

**Conclusion:**

These findings indicated that inhibition of oncogene SCD1 is required for targeting EGFR therapy in lung cancer.

## Background

Lung cancer is one of the leading causes of cancer-induced deaths worldwide, accounting for 1.59 million deaths annually [1]. Currently, lung cancer is pathologically classified into two main histologic types: non-small cell lung cancer (NSCLC) and small cell lung cancer (SCLC). NSCLC accounted for more than 85% of all lung cancers [2]. Due to large proportion of NSCLC patients, the locally advanced or metastatic stage has already been developed at the time of diagnosis, and the prognosis for advanced-stage disease is extremely unsatisfactory with an overall 5-year survival rate being 17%. More importantly, the recurrence rate remained particularly high as well, even though relevant treatments are performed in the early stage. Apart from the current surgery, radiotherapy and platinum-based chemotherapy, the inhibitors of EGFR by targeting tyrosine kinase inhibitors (TKIs) such as gefitinib or eloritinib contributed to clinical successes, but the patients would eventually acquire drug resistance [3–5].

The processes of cell proliferation, cell death and differentiation demanded a finely tuned sequential activation and deactivation of both biosynthetic and energy-generating metabolic pathways, especially the fatty acid metabolism [6]. The two major products included during this process were saturated fatty acids (SFA) and monounsaturated fatty acids (MUFA). Stearoyl-CoA desaturase (SCD) is a Δ9-fatty acyl CoA desaturase that catalyzes the formation of a double bond in the cis-delta-9 position of saturated fatty acyl CoAs, mainly the palmitoyl CoA and stearoyl CoA, producing palmitoleoyl CoA and oleoyl CoA, respectively, which is a rate-limiting enzyme responsible for MUFA synthesis [7, 8]. High rates of glycolysis and lipid biosynthesis are considered as the key hallmarks in carcinogenesis. Notably, SCD1 is highly expressed in oncogene-transformed lung fibroblasts and cancer cells, and is involved in sustaining rapid cancer cell proliferation, evading cell apoptosis, facilitating cancer cell initiation and malignant transformation in various types of cancers including leukemia [9], breast cancer [10], renal cancer [11], lung cancer [12], hepatocarcinoma [13] etc. We previously identified overexpression of SCD1 in tumor tissues of NSCLC patients and aberrantly high levels of SCD1 are predicted as a poor clinical outcome in patients with lung cancer [14]. However, the function and underlying mechanisms of SCD1 during the progression of NSCLC needs further elucidation.

Several pathways have been discovered to be involved in SCD1-regulated tumor characteristics, such as the AMP-activated protein kinase (AMPK) pathway [15], the phosphatidylinositol-3 phosphate kinase (PI3K)/Akt/mTOR pathway [16], and the epidermal growth factor receptor (EGFR) pathway [17]. These central signaling cascades are critical for the regulation of rapid lipid biosynthesis, growth and survival of cancer cells. Previous findings indicated that EGFR binds to and phosphorylates SCD1 at Y55 for maintaining the stability of SCD1 protein, increasing the MUFA levels to promote lung cancer growth [18]. The suppression of SCD activity with CVT-11127, a SCD inhibitor, impaired the ligand-induced phosphorylation of EGFR receptor, causing inactivation of its downstream targets such as Akt, ERK and mTOR. This in turn potentiated anti-proliferative effect of Gefitinib in vitro [17]. Likewise, we found that overexpression of SCD1 in lung cancer cells could abrogate Gefitinib-induced apoptosis and inhibit cell vitality. Hence, targeting EGFR signals is regarded as a promising strategy for the treatment of NSCLC [12]. SCD1 modulates the activity of EGFR/Akt/ERK signaling pathway to control cancer cell metabolism, proliferation and survival [17]. Using shRNA-SCD1 or SCD1 inhibitor, Zhang et al. have also reported that EGFR-stimulated cancer growth depends on SCD1 activity. EGFR stabilizes SCD1 through Y55 phosphorylation, thereby up-regulating MUFA synthesis to promote lung cancer growth [19]. Moreover, it has been revealed that SCD1 increases colorectal cancer (CRC) progression through promoting epithelial-mesenchymal transition (EMT), promoting metastasis of CRC cells through MUFA production and suppressing PTEN in response to glucose [20]. Therefore, in this study, we focused on evaluating the function of SCD1 in the treatment of EGFR-targeted lung cancer.

## Materials

### Cell lines and reagents

The lung cancer cell lines A549, H838 and H1573, and human bronchial epithelial cells BEAS-2B were purchased from the cell bank of the Chinese academy of sciences (Shanghai, China). The cells were cultured in Dulbecco’s Modified Eagle’s medium (DMEM) supplemented with 10% FBS (Life Technologies, USA), ampicillin and streptomycin at 37 °C in 5% CO_2_ conditions. The recombinant vector pcDNA3.1-SCD1 with overexpression of SCD1 and negative control were conducted by GenePharma (Shanghai, China). Antibodies against SCD1, Caspase-3, phosphorylated PI3K (p-PI3K), p-EGFR and Akt were purchased from Abcam. A939572 (HY-50709) was purchased from MedChem Express (USA). Gefitinib (ZD1839) was purchased from Apexbio (USA).

### Real-time PCR

To estimate the levels of SCD1 in lung cancer samples or cell lines, total RNA from cell lines was extracted by using Trizol reagent (Invitrogen). According to the standard protocol, quantitative real-time RT-PCR (qRT-PCR) was performed, and the relative levels of SCD1 were normalized to GAPDH, which acts as a reference gene.

### Cell transfection and CCK-8 assay

The A549 and H1573 cell lines were seeded into a well of 96-well plate and were cultured to about 80% confluence, and then were transfected with pcDNA3.1-SCD1 or negative control for an indicated time period at a concentration of 1 ng/ml by Lipofectamine 2000 (Invitrogen, USA) according to the manufacturer’s instructions. The cells were incubated with different concentrations of Gefitinib (0, 5, 10, 20, 40, and 80 μM) or with A939572 (1 nM). After incubation, CCK-8 solution (10 μl) was added to each well and the cultures were incubated at 37 °C for 90 min to determine the cell viability. The OD values in different wells were detected with a microplate reader at 450 nm.

### Western blots

According to the manufacturer’s instructions, the whole cell protein extracts from cell lines or lung cancer tissues were prepared, separated on 4–15% polyacrylamide gel, and then transferred onto the nitrocellulose membranes (Amersham Biosciences). The membranes were blocked in 5% non-fat milk in TBST buffer (Tris Buffer Saline containing 0.1% Tween-20) for 1 h at room temperature, followed by incubation with primary antibodies to SCD1, p-PI3K, p-EGFR, p-AKT and caspase-3 overnight at 4 °C. After washing with TBST buffer, the blots were then incubated with HRP-conjugated secondary antibody for 2 h at room temperature. The blots were washed with TBST buffer and visualized using the ECL-Plus reagent (Millipore, Billerica, MA, USA). GAPDH was used as the loading control.

### Immunofluorescence

After treatment with Gefitinib and A939572, the cells were fixed in 4% paraformaldehyde for 15 min and then washed three times with PBS. Then the cells were blocked with 5% non-fat milk in 0.1% Triton X-100 at room temperature for 1 h, followed by anti-E-cadherin and N-cadherin primary antibodies (Sigma-Aldrich, St. Louis, MO, USA) for 1 h. Next, the cells were incubated with Alexa Fluor 488 or 594-labeled secondary antibodies for 1 h and washed with PBS. DAPI counterstain was used to demonstrate the nuclei. Stained cells were visualized using immunofluorescence microscopy.

### The apoptosis analysis

After the treatment with Gefitinib and A939572, the cells were fixed in cold 70% ethanol at − 20 °C for 2 h. The cells were then treated with 10 mg/ml RNase and stained with 2 μl of annexin V mixed with 2 μl of Propidium iodide (PI, eBioscience) according to the manufacturer’s instructions. The cells were quantified by using flow cytometry on a FACS Calibur instrument.

### Transwell assay

The A549 and H1573 cell lines were transfected with SCD1 and treated with Gefitinib and A939572. In the migration assay, 2 × 10^4^ cells were in the upper chamber of a non-coated transwell insert. In the invasion assay, the upper chamber of the transwell inserts were coated with Matrigel, and the tumor cells were plated in the upper chamber of the Matrigel-coated transwell insert. Cells that did not migrate or invade were removed using a cotton swab, followed by staining using crystal violet and counting under an inverted microscope. Five random views were selected to count the cells.

### Tumor model

2 × 10^6^ A549 cells transfected with lentivirus vector of pcDNA3.1-SCD1 or negative control were subcutaneously injected into the rear flank of nude mice (6 per group). The nude mice were orally fed with A939572 (30 mg/kg, p.o. twice daily) or Gefitinib (100 mg/kg, p.o. 3 days apart) for 20 days, and then the tumor volume (mm^3^) was recorded. The tumor sizes were measured three days apart and the tumor volumes were calculated using the formula: V (cm^3^) = width^2^ (cm^2^) * length (cm)/2. The animal study was approved by the institutional animal research committee of The First Affiliated Hospital of Guangzhou Medical University.

### Immunohistochemistry

Expression levels of Ki-67 in paraffin-embedded tissues of mouse lungs were detected by immunohistochemistry as previously described [21]. The tissues were treated with anti-human Ki-67 (Cell Signaling Technology, Danvers, MA) monoclonal antibodies. Images were acquired using a fluorescence microscope (DM2000; Leica Micro-systems, Wetzlar, Germany).

### Statistical analyses

The Statistical Package for Social Sciences version 16.0 (SPSS 16.0, SPSS Inc., Chicago, IL, USA) and the Prism statistical software package (Version 5.0, Graphpad Software Inc.) were used. Unpaired t-tests or Mann–Whitney U tests were used to compare the two groups, and one-way ANOVA was used for multiple group comparisons. *p *< 0.05 was considered to be statistically significant. All experiments were performed at least three times.

## Results

### Overexpressed SCD1 promotes the resistance of EGFR inhibitor-based therapy

**Fig. 1:**
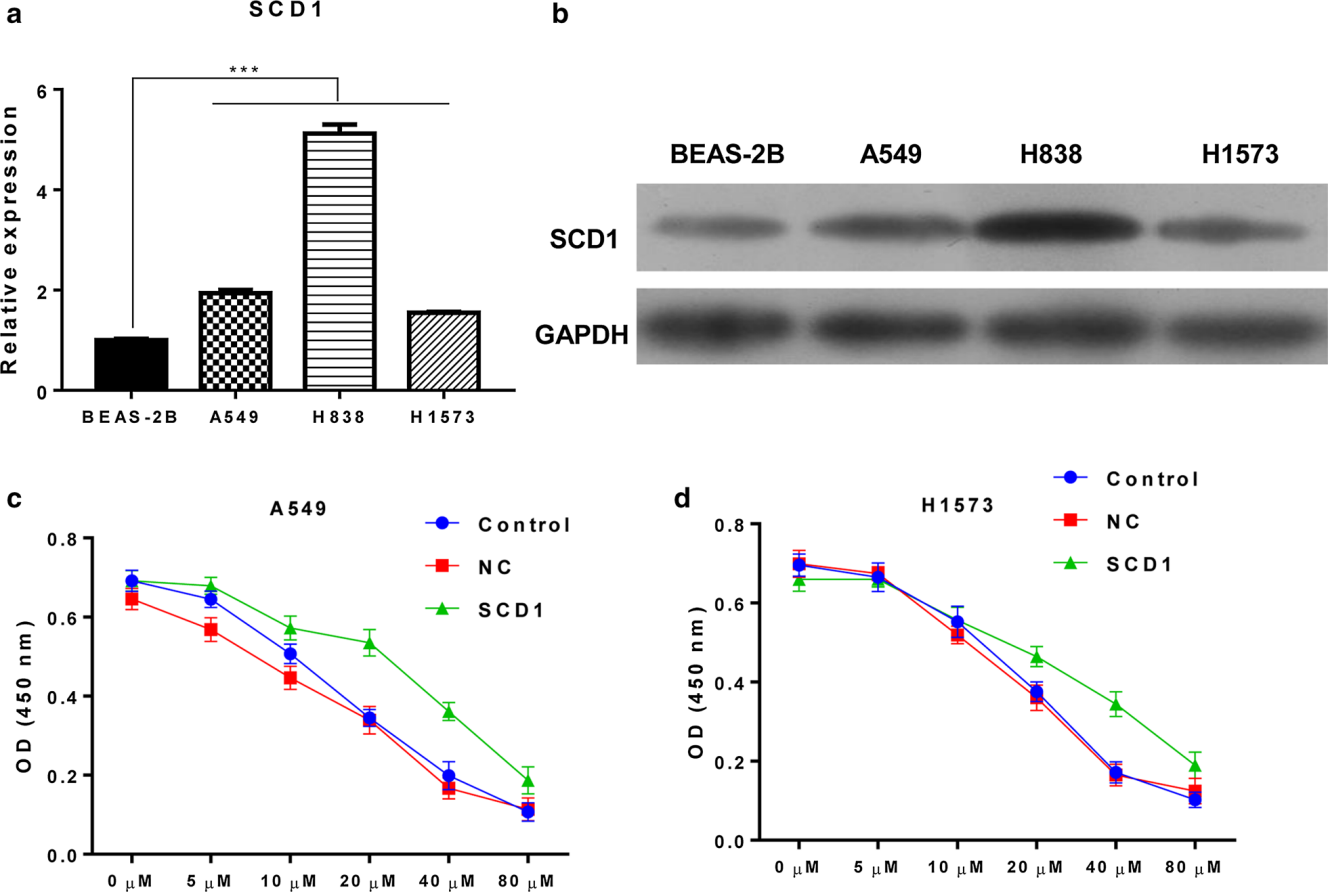
Cells with overexpressed SCD1 were resistant to Gefitinib. **a**, **b** The expression of SCD1 in five lung cancer cell lines A549, H838, H1573 and one normal human bronchial epithelial cells BEAS-2B was analyzed. **c**, **d** The cell vitality of A549 and H1573 with or without SCD1 overexpression was assessed after treatment with different doses of Gefitinib (0, 5, 10, 20, 40, and 80 μM) for 72 h. *NC* negative control.***p < 0.001, data is presented as mean ± sd

The up-regulation of SCD1 in lung cancer cell lines A549, H838 and H1573 should be confirmed. The results indicated that the mRNA and protein levels of SCD1 were remarkably increased in cancer cell lines when compared to human bronchial epithelial cells BEAS-2B (Fig. 1a, b). Based on this, we have chosen A549 and H1573 cell lines for further overexpression assay due to their relatively lower expression of SCD1 in the four cell lines. To determine the role of SCD1 in EGFR inhibitor-based therapy, the two cell lines were treated with different concentrations of Gefitinib (0, 5, 10, 20, 40, and 80 μM) for 72 h. The results of statistical analysis showed that the IC50 of Gefitinib for A549 and H1573 cell lines with overexpressed SCD1 (42 μM and 40 μM) were significantly higher than that in the control and negative control (NC) groups (21 μM and 18 μM) (Fig. 1c, d). These data indicated that SCD1 promotes the resistance of Gefitinib in lung cancer.

### SCD1 is required for Gefitinib-induced cytotoxicity in lung cancer

**Fig. 2:**
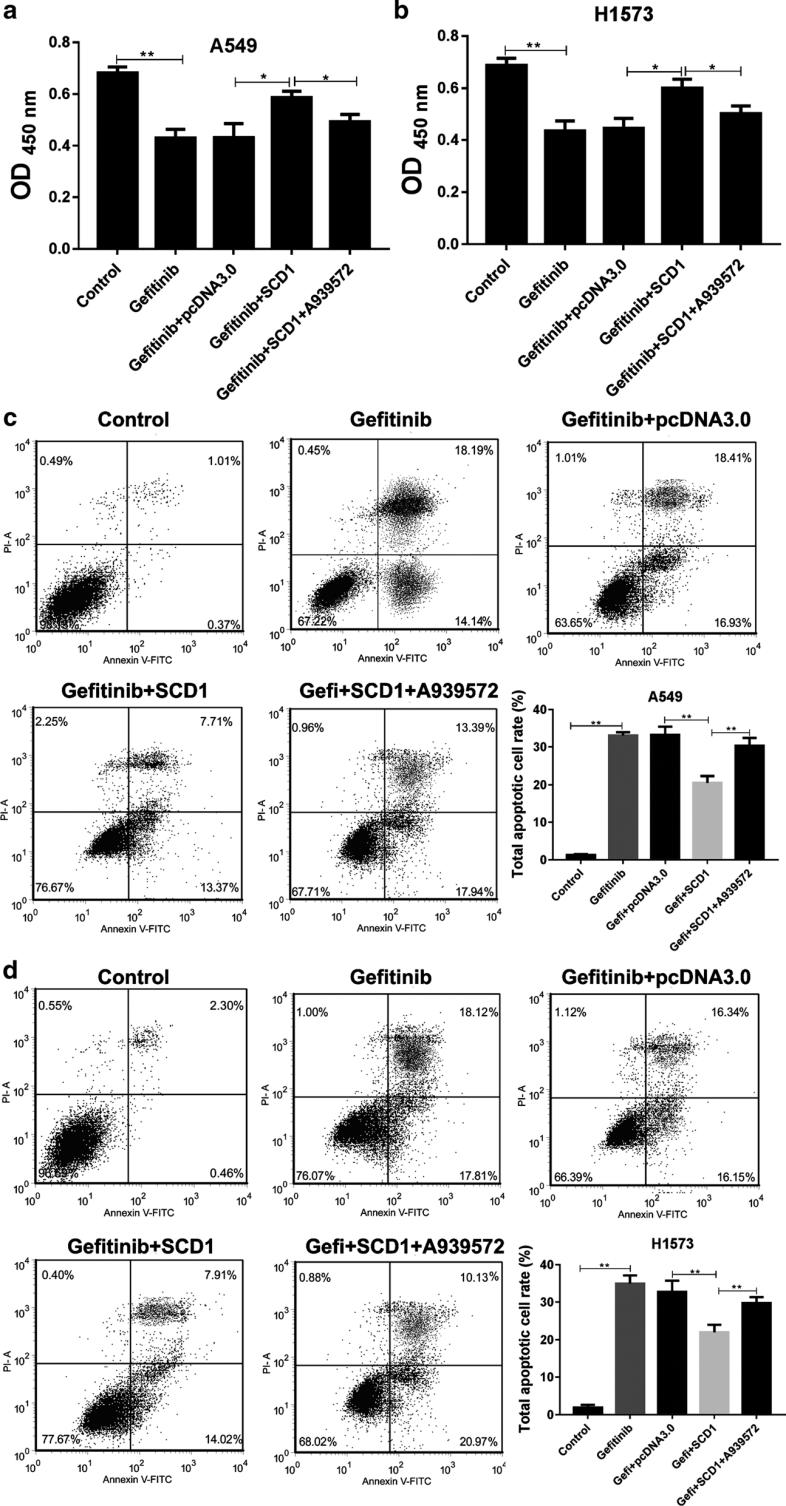
SCD1 inhibits Gefitinib-induced cytotoxicity in lung cancer. **a**, **b** The cell vitality of A549 and H1573 cells with or without SCD1 overexpression was assessed by CCK-8 assay after treatment with Gefitinib (20 μM) and A939572 (1 nM) for 48 h. Meanwhile, the total apoptosis of A549 (**c**) and H1573 cells (**d**) was also determined by flow cytometry. *p < 0.05, **p < 0.01, data is presented as mean ± sd

To investigate the role of SCD1 during the treatment of Gefitinib, we used SCD1 inhibitor, A939572 (1 nM). The results showed that the cell vitality was inhibited by Gefitinib (20 μM), but this inhibition was conversed when the two cell lines were forced to express SCD1. More importantly, the addition of SCD1 inhibitor A939572 could abrogate the SCD1 activity and restore the cytotoxicity of Gefitinib in A549 and H1573 cell lines (Fig. 2a, b). Similarly, the cell apoptosis was also estimated. Flow cytometry results showed that the Gefitinib treatment increased the apoptosis of A549 and H1573 cell lines. In contrast, the overexpression of SCD1 helped the tumor cells from Gefitinib-induced apoptosis. However, the rescuing role of SCD1 was abrogated by A939572, indicating that SCD1 protects the cells from Gefitinib-induced apoptosis (Fig. 2c, d).

### SCD1 inhibition restores Gefitinib-impaired migration and invasion of lung cancer cells

**Fig. 3:**
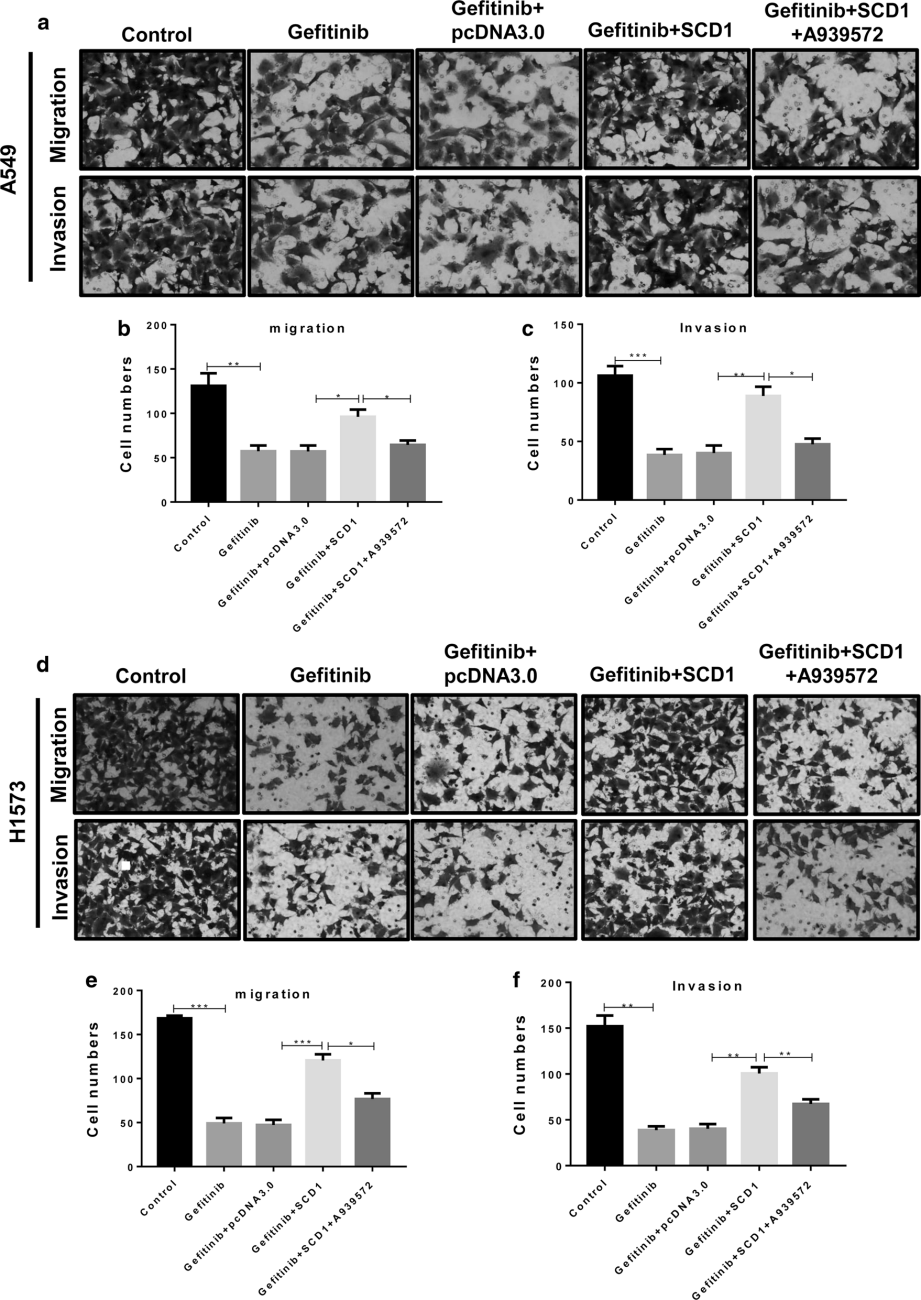
SCD1 re-activates Gefitinib-impaired migration and invasion in lung cancer. The A549 and H1573 cells with or without SCD1 overexpression were treated with Gefitinib (20 μM) and A939572 (1 nM), and the migration and invasion of the cells were assessed by Transwell assay. *p < 0.05, **p < 0.01, data is presented as mean ± sd

Due to the pro-metastatic effects of EGFR signals in cancer cells, other than the cytotoxicity induced by Gefitinib, the function of SCD1 in the ability to migrate and invade A549 (Fig. 3a–c) and H1573 cell lines (Fig. 3d–f) was estimated. The results revealed that Gefitinib significantly repressed the migration and invasion of two cell lines, and was attenuated by SCD1 overexpression. These results suggested that SCD1 might increase the migratory and invasive ability, although the EGFR signals were defective. Indeed, when the SCD1 inhibitor A939572 was added, the pro-metastatic effects were remarkably suppressed in A549 and H1573 cell lines. Thus, SCD1 was required for EGFR signal-activated metastasis.

### SCD1 activates EGFR/PI3K/AKT signals and up-regulated EMT phenotype

**Fig. 4:**
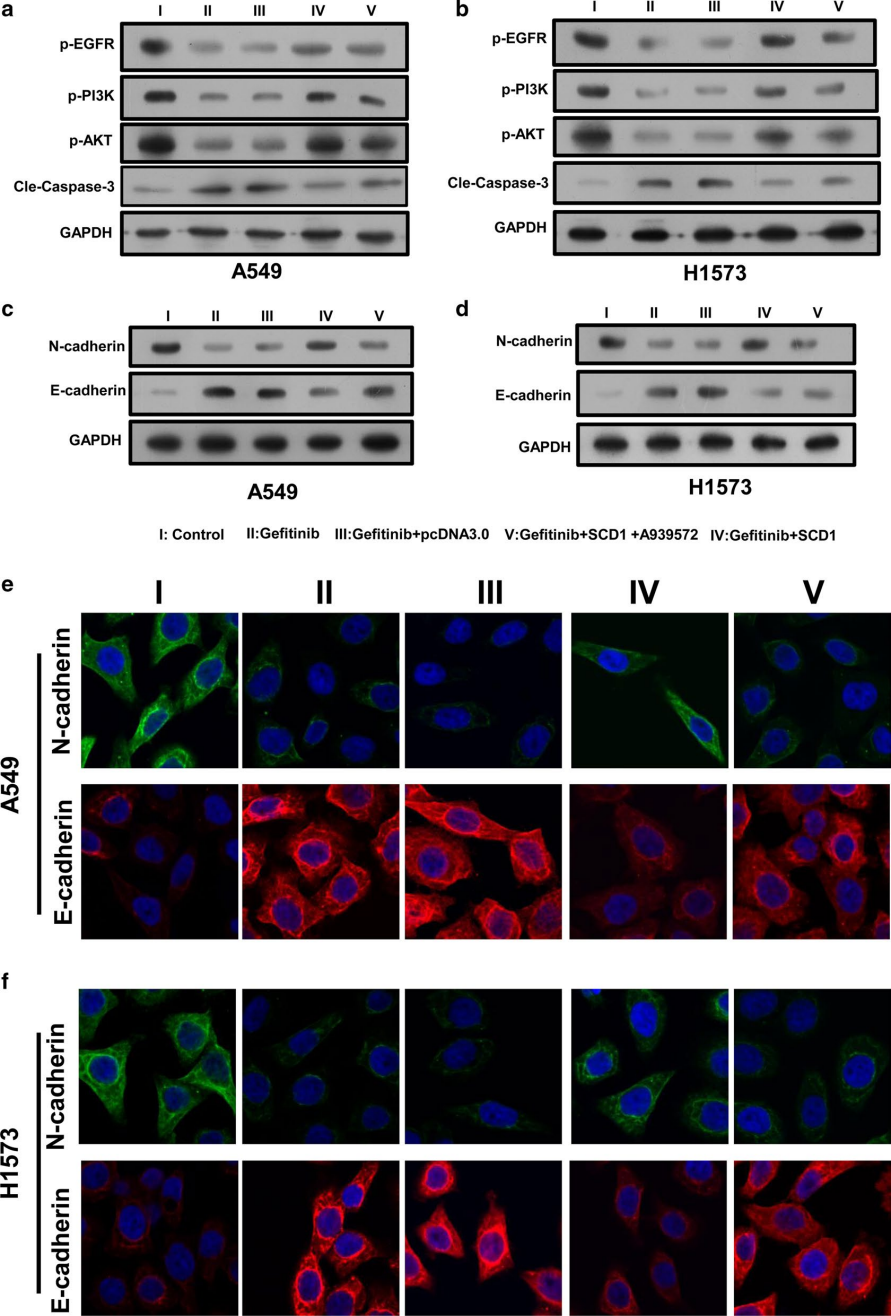
SCD1 activates EGFR/PI3K/Akt signaling and EMT phenotype of lung cancer cells. After treatment with Gefitinib (20 μM) and A939572 (1 nM), **a**, **b** the phosphorylation of EGFR/PI3K/AKT signaling pathway and the expression of caspase-3 were determined by western blotting. The expression of E-cadherin and N-cadherin was analyzed by western blotting (**c**, **d**) an immunofluorescence (**e**, **f**)

Accumulated evidence has demonstrated that SCD1 promotes the activation of EGFR/PI3K/AKT signaling for cell survival, proliferation and chemotherapy resistance in many cancer types. Thus, the activation of EGFR/PI3K/AKT signaling was analyzed. The results found that the lung cancer cells had high levels of activated EGFR/PI3K/AKT signaling. Gefitinib treatment could impair the phosphorylation of EGFR/PI3K/AKT signaling. However, the cells with overexpressed SCD1 restored the phosphorylation of EGFR/PI3K/AKT signaling (Fig. 4a, b). The addition of A939572 down-regulated the availability of *SCD1*, abrogating this process to reduce the resistance to Gefitinib. This in turn led to the activation of caspase-3-dependent apoptosis via cleavage of caspase-3 (Fig. 4a, b).

These pathways are critical for the initiation and aggravation of metastasis via regulation of EMT phenotype. Thus, the EMT phenotype including mesenchymal phenotype N-cadherin and the epithelial phenotype E-cadherin were also determined in A549 (Fig. 4c, e) and H1573 cell lines (Fig. 4d, f). The results showed that Gefitinib could inhibit the EMT process via increased E-cadherin and decreased N-cadherin expression, but SCD1 expression promoted the EMT process via re-activation of EGFR/PI3K/AKT signaling, leading to the up-regulation of migration and invasion.

### SCD1 is required for anti-tumor effects of Gefitinib in vivo

**Fig. 5:**
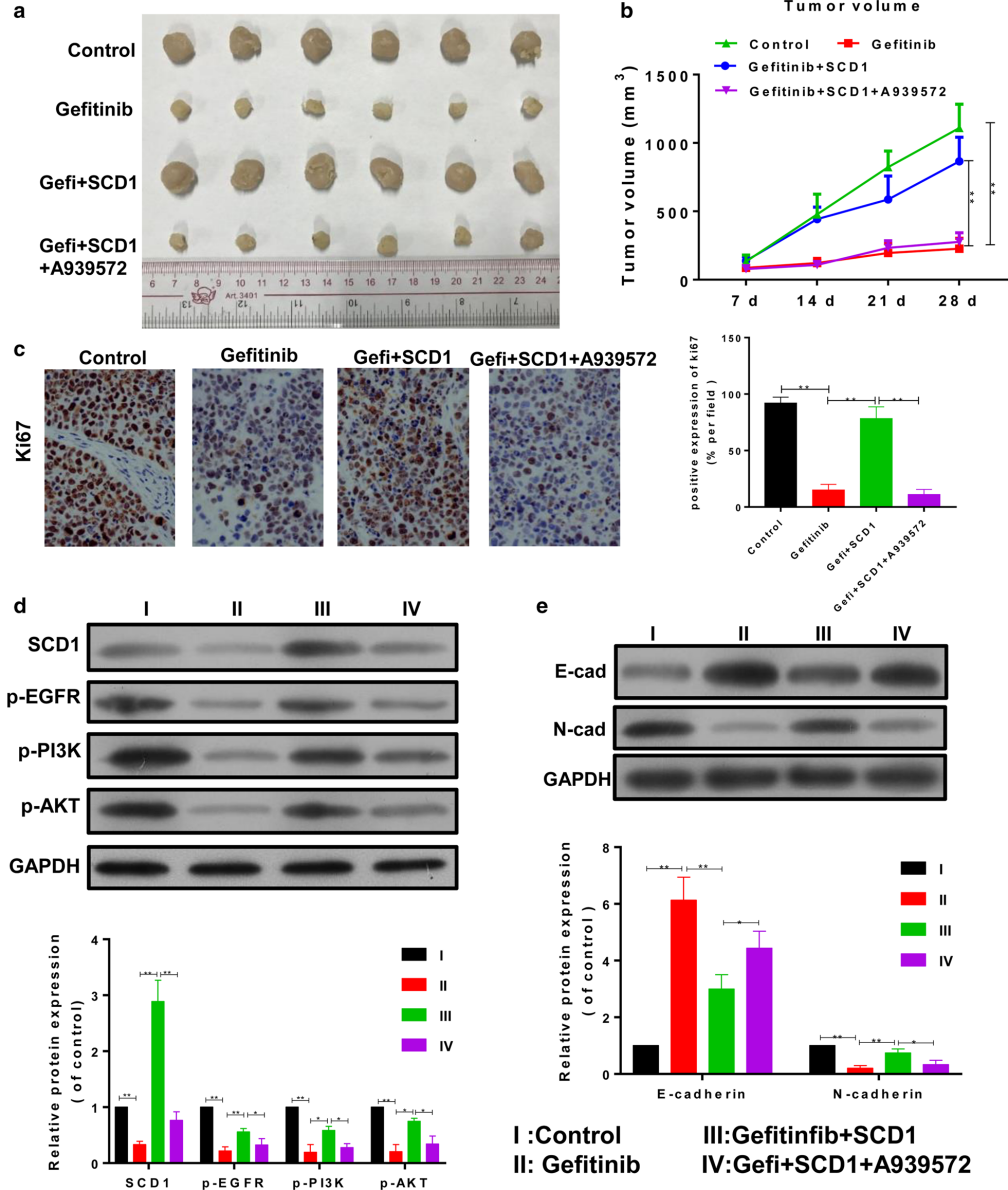
SCD1 is required for Gefitinib-induced tumor regression in vivo. Human A549 cells were transfected with lentiviral vector of pcDNA3.1-SCD1 or negative control and subcutaneously injected into nude mice. **a**, **b** The tumor size and the mean tumor size (mm^3^) were analyzed. **c** The expression of Ki-67 was analyzed in tumor tissues. **d** The expression of SCD1 was analyzed in tumor tissues. The EGFR/PI3K/AKT signaling, and **e** the EMT phenotype in tumor tissues were also determined by western blotting. *p < 0.05, **p < 0.01, ***p < 0.001, data is presented as mean ± sd

We herein confirmed the role of SCD1 in Gefitinib treatment in vivo. We established a xenograft model of human A549 and the nude mice were administered with conditional tumor cells that are SCD1 overexpressed or negative control. The nude mice were orally fed with A939572 (30 mg/kg, p.o.) or Gefitinib (100 mg/kg, p.o. 3 days apart), and then the tumor volume (mm^3^) was recorded. The results showed that Gefitinib promoted tumor regression with decreased tumor volume, but the mice with SCD1 overexpression abrogated the anti-tumor role of Gefitinib and promoted the tumor growth with increased tumor volume, which was then inhibited by SCD1 inhibitor (Fig. 5a, b). The expression of proliferation index Ki-67 was consistent with the tumor volume in each group (Fig. 5c). Mechanistically, the expression of SCD1 in tumor tissues was inhibited by Gefitinib and A939572, resulting in the decreased activity of SCD1 in tumor tissues (Fig. 5d). In parallel to this, the mice with SCD1 overexpression showed re-activation of EGFR/PI3K/AKT signaling, which was also inhibited by A939572 (Fig. 5d). Similar to the in vitro results, the EMT phenotype in vivo was also modulated by SCD1. These findings suggested that SCD1 was required for inducing the anti-tumor effects of Gefitinib in vivo (Fig. 5e).

## Discussion

In China, lung cancer is still the leading cause of cancer deaths in 2015. Cytotoxic chemotherapy as a conventional treatment of NSCLC has been gradually changed by the development of immunotherapeutic agents [22]. Clinical data showed that targeted therapy with EGFR inhibitor, TKIs, showed no significant prolongation in the overall survival and single TKI treatment is not enough for the treatment of NSCLC patients due to potential cause of drug resistance [23]. SCD1 is a critical rate-limiting enzyme during the fatty acid metabolism pathway and belongs to a family of fatty acyl desaturases [7]. SCD1 is universally present in all mammalian cells, with the highest levels in the brain, liver, heart and lung. SCD1 is highly expressed in oncogene-transformed fibroblasts and in cancer cells [24]. The elevated levels of SCD1 are associated with poor prognosis in breast cancer patients [25]. Of note, our previous study also showed the up-regulated expression of SCD1 in lung cancer tissues, which was correlated with shorter overall survival time of patients with NSCLC [14]. In this study, we also confirmed that the expression of SCD1 remained to be higher in lung cancer cell lines than that in normal lung epithelial cells. SCD1 in lung cancer controls cell cycle progression, apoptosis, proliferation and cancer stem cells [12, 19]. Several evidences have demonstrated that the SCD1 activity levels control the oncogenic phenotype by regulating the signaling platforms of critical relevance in carcinogenesis, especially the EGFR/PI3K/Akt, mTORC and AMPK signaling pathways [26], suggesting that SCD1 might be involved in the regulation of EGFR TKIs drug resistance though PI3K/Akt signaling pathway. In this scenario, we explored the effect and mechanism of SCD1 in the EGFR inhibitor-based therapy for NSCLC.

We herein found that the lung cancer cells with overexpressed SCD1 showed high resistance to EGFR inhibitor-induced cytotoxicity with high IC50 values than that with relatively low expressed SCD1. This suggested that the activity of SCD1 was inhibited during treatment with TKIs. Also, in this study, the SCD1-overexpressed lung cancer cells were treated with SCD1 inhibitor A939572 during Gefitinib treatment. We found that A939572 could significantly impair the phosphorylation of EGFR/PI3K/Akt signaling, and parallelly suppressed the cell vitality, apoptosis in vitro and tumor growth in vivo of lung cancer cells to restore the sensitivity to Gefitinib.

The correlation between SCD1 and metastasis was been found. Mauvoisin et al. found that silencing SCD1 expression in breast cancer cells has no effect on cell viability, but inhibited GSK3 phosphorylation and β-catenin translocation to the nucleus, leading to impaired EMT-like behavior of the cells. siRNA SCD1 showed a higher level of expression of the epithelial marker E-cadherin and a lower level of expression of mesenchymal marker N-cadherin compared to the control cells [10]. Liu et al. also revealed the pro-metastatic effects of SCD1 in lung cancer by targeting SCD1, which effectively inhibited lung metastasis and prolonged the overall survival of mice [27]. This was consistent with our findings in lung cancer. Although Gefitinib inhibited the initiation of EMT, we reported that SCD1 overexpression re-activated the EMT phenotype, which was inhibited by EGFR inhibition, but when the SCD1 activity was repressed by its inhibitor, the pro-EMT function was remarkably abrogated. These findings were also confirmed in tumor tissues in vivo.

## Conclusions

Our data demonstrated that overexpressed SCD1 in lung cancer cells is a disadvantage for EGFR-targeting cancer therapy of NSCLC. SCD1 impaired Gefitinib-induced cytotoxicity and re-activated the EMT phenotype both in vitro and in vivo. The addition of SCD1 inhibitor could converse these effects via inhibiting SCD1-dependent phosphorylation of EGFR/PI3K/Akt signaling in lung cancer.

## Abbreviations

SFAs: saturated fatty acids
MUFAs: monounsaturated fatty acids
SCD: stearoyl-CoA desaturases
EMT: epithelial to mesenchymal transition
NSCLC: non-small cell lung cancer
TKIs: tyrosine kinase inhibitors
PI3K: phosphatidylinositol-3 phosphate kinase
EGFR: epidermal growth factor receptor
